# The aspartate superpathway in gut microbiota-related metabolic pathways mediates immune cell protection against COPD and IPF: a Mendelian randomization analysis

**DOI:** 10.18632/aging.206250

**Published:** 2025-05-15

**Authors:** Lei Chen, Haoyan Chen, Qin Li, Jun Ma, Yanzhi Feng, Shenghua Zhang, Yu Han, Jie Pan, Mingjiong Zhang, Kai Sun, Shuangshuang Wu

**Affiliations:** 1 Department of Geriatrics, Jiangsu Key Laboratory of Geriatrics, The First Affiliated Hospital with Nanjing Medical University, Nanjing, China; 2 Center of Molecular and Cellular Oncology, Yale School of Medicine, New Haven, CT 06510, USA; 3 Department of Respiratory Medicine, Affiliated Nantong Hospital of Shanghai University (The Sixth People’s Hospital of Nantong), Nantong, China; 4 The First Affiliated Hospital of Nanjing Medical University, Nanjing, China; 5 Department of Rheumatology and Immunology, Liyang Branch of Jiangsu Province Hospital, Liyang, China

**Keywords:** Mendelian randomization, gut microbiota, idiopathic pulmonary fibrosis (IPF), chronic obstructive pulmonary disease (COPD), immune cells

## Abstract

Background: Both genetic and environmental factors can influence idiopathic pulmonary fibrosis (IPF) and chronic obstructive pulmonary disease (COPD) development. The gut microbiota plays crucial roles in maintaining tissue homeostasis. Dysregulation of the gut microbiota can result in disease. However, whether the alteration of the gut microbiota influences IPF and COPD remains unknown.

Research Question: What is the causal relationship between IPF, COPD and the gut microbiota-related metabolic pathways? What are the potential intermediate mediators in this relationship?

Study Design and Methods: Intersect the gut microbiota and its metabolic pathways associated with IPF and COPD. Utilizing summary data from GWAS in public databases, a two-sample Mendelian randomization (MR) analysis was conducted on the gut microbiota-related metabolic pathway, the aspartate superpathway, in relation to IPF and COPD. Furthermore, we employed a two-step MR to quantify the proportion of influence mediated by monocytes and cDCs on the aspartate superpathway in relation to IPF and COPD.

Results: The MR analysis found that the aspartate superpathway decreased the risk of developing IPF and COPD. Monocytes and cDCs acted as intermediary substances, participating in this with influence proportions of 7.88% and 6.27%, respectively.

Interpretation: There is a causal link between the gut microbiota-related metabolic pathway, the aspartate superpathway, and IPF and COPD, where the influence is partially mediated by monocytes and cDCs. In clinical practice, we increase the focus on gut microbiota-mediated immune cells in relation to IPF and COPD.

## INTRODUCTION

The human gastrointestinal tract hosts an extensive community of microorganisms, primarily consisting of bacteria and fungi, collectively known as the gut microbiota [1]. This microbial community comprises approximately 40 trillion organisms, possessing a genetic repertoire about 150 times larger than that of the human host [2]. Consequently, the gut microbiota has been dubbed “the second brain,” owing to its pivotal roles in food digestion, synthesis of essential vitamins, and modulation of the immune system [3]. This intricate ecosystem profoundly influences the internal environment of the human body, playing a crucial role in maintaining homeostasis. The emergence of the Gut-lung axis concept highlights the deep interplay between intestinal dysbiosis and gastrointestinal function with distal organ function, particularly the lungs [4].

Chronic Obstructive Pulmonary Disease (COPD) and Idiopathic Pulmonary Fibrosis (IPF) represent the most prevalent chronic respiratory ailments that particularly afflict the elderly demographic [5]. The global prevalence of COPD and IPF poses significant public health challenges, given their profound impact on airway structure alteration and heightened mortality rates among older adults [6]. Although the precise etiology and pathogenesis of COPD and IPF remain incompletely elucidated, their development is believed to be influenced by a confluence of genetic and environmental factors [7]. Mounting evidence underscores the growing association between gut microbiota composition and chronic respiratory disorders [8].

Gut microbiota can influence the immune system both locally, through cell-cell interactions, and systemically, through their metabolites. Consequently, the aberrant alteration of gut microbiota can lead to the onset and progression of disease [9]. For example, gut microbial dysbiosis has been implicated in the pathogenesis of lung diseases through various immune-mediated pathways, including modulation of T helper 17 cell (Th17) responses, CD8 T-cell activity, and interleukin-13 (IL-13), interleukin 25 (IL-25), and prostaglandin E2 production [10]. Moreover, disruption of intestinal barrier integrity facilitates the translocation of gut microbiota-derived components into the bloodstream, subsequently initiating chronic inflammation and fibrotic processes in the lungs. These insights offer novel therapeutic avenues and mechanistic insights for the understanding and intervention of COPD and IPF [11].

Notably, monocyte-derived macrophages and endothelial cells have emerged as key players in the fibrotic cascade underlying IPF pathogenesis [12]. Clinical studies have underscored the prognostic value of monocyte profiles in IPF patients, while single-cell RNA sequencing of lung tissues has unveiled the considerable heterogeneity of monocyte-derived macrophages in IPF pathophysiology [13]. Similarly, dendritic cells are one of the major antigens - presenting entities. Among them, cDC cells can be classified into two types: cDC1 and cDC2. cDC1 cells mainly activate CD8+ T cells, initiate cellular immunity, and play a prominent role in aspects such as anti - tumor immunity. cDC2 cells are more likely to activate CD4+ T cells, participate in humoral immunity, and play an important role in processes including anti-parasitic immunity and immune regulation. Studies have found that the expression pattern of dendritic cells in patients with chronic obstructive pulmonary disease is altered in comparison to healthy individuals, indicating that dendritic cells play a crucial role in chronic respiratory diseases [14].

In our investigation, we employed a two-sample, bidirectional Mendelian Randomization approach to delineate the causal relationships between gut microbiota-related metabolic pathways and the onset of IPF and COPD. We uncovered that immune cell interactions serve as an important mediator in this causality. This integrative approach provides novel insights into the pathogenesis of IPF and COPD, paving the way for future innovative therapeutic strategies against these diseases.

## MATERIALS AND METHODS

### Study design

**Figure 1:**
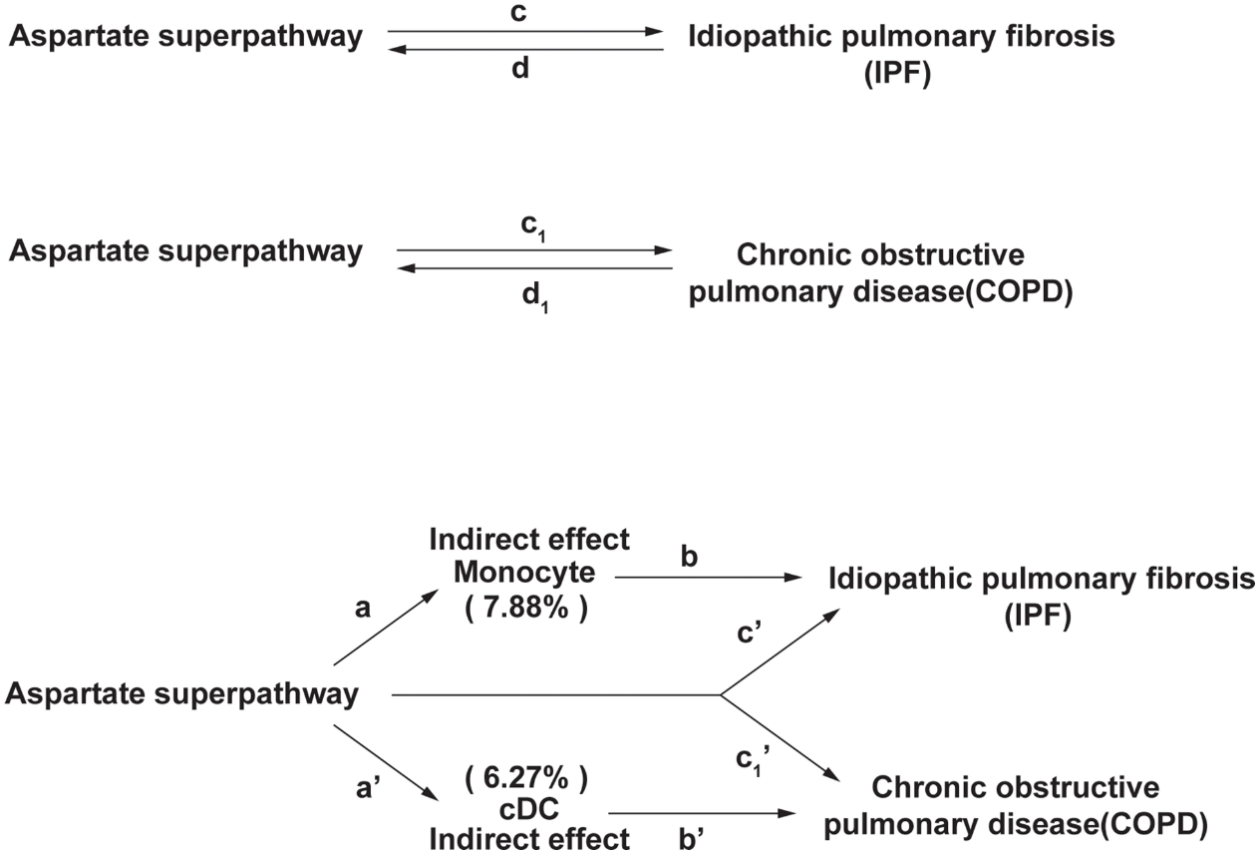
**The experimental design of this study.** In IPF, the indirect effect = a×b, and the direct effect is c’. In COPD, the indirect effect = a’×b’, and the direct effect is c1’. The proportion of the mediating effect is the indirect effect divided by the total effect.

We conducted a two-sample, bidirectional Mendelian Randomization (MR) study to investigate the causal associations between potential metabolic pathways of gut microbiota and Idiopathic Pulmonary Fibrosis (IPF) and Chronic Obstructive Pulmonary Disease (COPD). The study consisted of several sequential steps (Figure 1):

1. Screening of metabolic pathways associated with gut microbiota.
2. Two-sample MR analysis to examine the relationships with IPF and COPD.
3. Exploration of immune cells as mediators to assess causality between bacterial genus metabolic pathways and the diseases.
4. Analysis of the proportion of mediated effects.

### Source of GWAS data

GWAS data on the gut microbiome were sourced from a study involving 7738 Dutch participants, investigating 207 taxonomic units and 205 pathways representing microbial composition and function [15]. IPF-associated genetic variants were obtained from a study published in The Lancet involving 451,025 patients [16], while COPD-related summary statistics were obtained from published studies [17]. Pooled GWAS data related to immune cell traits were obtained from the NHGRI-EBI GWAS Catalog.

### Selection of instrumental variables (IVs)

IVs were selected based on their close relationship with the exposure factor, exclusive effect on the outcome through the exposure factor, and independence from any confounders. IVs were chosen at a significance level of 1 × 10^-5,^ and weak and chained imbalance variables were removed. A total of 11 IVs related to the aspartate superpathway, 48 COPD-related IVs, 18 IPF-related IVs, 32 cDC-related IVs, and 22 monocyte-related IVs were selected.

### Mendelian randomization

Two-sample bidirectional MR was employed to assess the causal links between the aspartate superpathway and IPF/COPD. Mendelian randomization commonly employs the following five algorithms: 1) MR Egger: It is a Mendelian randomization analysis method that incorporates the Wald ratio into meta - regression. It can detect and correct horizontal confounding factors, provide evidence of direct confounding through the intercept, and estimate the causal effect adjusted for pleiotropic effects in any direction. However, it has relatively low statistical power. 2) Weighted median: It is one of the Mendelian randomization analysis methods. It assigns greater weights to valid instrumental variables. Even if some instrumental variables are invalid, it can still provide valid estimates of the causal effect. When 50% of the instrumental variables are invalid, a consistent estimate can still be obtained. 3) Inverse variance weighted (IVW): It is the main analysis method in Mendelian randomization. It assumes that all instrumental variables are valid, uses the reciprocal of the outcome variance as the weight for fitting, and does not consider the intercept term. When all genetic variations meet the instrumental variable assumptions, it can provide a consistent estimate of the causal effect. 4) Simple mode: It is a mode - based Mendelian randomization estimation model. It can cluster SNPs with similar causal effects and return the estimated causal effect of most clustered SNPs. 5) Weighted mode: It is also a mode - based estimation model. It usually has a relatively low bias and type - I error rate, but its ability to detect causal effects is also relatively low. In Mendelian randomization analysis, it can estimate the causal effect by comprehensively considering the weights of different SNPs.

### Mediated effects analysis

Mediation analyses were conducted to examine the influence of gut microbiota metabolic pathways and immune cells on IPF and COPD. The overall effect was calculated as the sum of the indirect and direct effects. The indirect effect represented the pathway’s impact on the disease through immune cells, while the direct effect denoted the pathway’s direct influence on the disease.

### Mediation effect for idiopathic pulmonary fibrosis (IPF)

Let the aspartate superpathway be the exposure factor *X*, monocytes be the mediator variable *M*, and idiopathic pulmonary fibrosis (IPF) be the outcome variable *Y*.

Effect of *X* on *M* : *a* represents the effect of the aspartate superpathway on monocytes, i.e., *M* = a*X* + *ε*1; Effect of *Y* on *M* : *b* represents the effect of monocytes on idiopathic pulmonary fibrosis, i.e., *Y* = b*M* + *ε*2; Mediation effect (Indirect effect): The mediation effect is *a*×*b*, and the proportion of the indirect effect is 7.88%; Direct effect: *c*' represents the direct effect of the aspartate superpathway on idiopathic pulmonary fibrosis, i.e., *Y* = *c*'*X* + b*M* + *ε*3.

### Mediation effect for chronic obstructive pulmonary disease (COPD)

Let the aspartate superpathway be the exposure factor *X*, cDC be the mediator variable *M*, and chronic obstructive pulmonary disease (COPD) be the outcome variable *Y*.

Effect of *X* on *M*: *a*' represents the effect of the aspartate superpathway on cDC, i.e., *M* = a'*X*+*ε*1; Effect of *Y* on *M* : *b*' represents the effect of cDC on chronic obstructive pulmonary disease, i.e., *Y* = b'*M* + *ε*2; Mediation effect (Indirect effect): The mediation effect is *a* × *b*, and the proportion of the indirect effect is 6.27%; Direct effect: *c*1' represents the direct effect of the aspartate superpathway on chronic obstructive pulmonary disease, i.e., *Y* = *c*1' *X* + b'*M* + *ε*3.

### Statistical analysis

Statistical significance was considered at *p < 0.05*. Student’s t-tests were utilized for comparisons between groups. All data analysis was performed using R Foundation version 4.2.0.

### Consent for publication

All authors have agreed to publish this article.

### Availability of data and material

All data analyzed during this study are included in the article.

## RESULTS

### Screening of metabolic pathways in the gut microbiota

Utilizing the biological data repository described above, we conducted an analysis to correlate gut microbiota composition with Idiopathic Pulmonary Fibrosis (IPF) and Chronic Obstructive Pulmonary Disease (COPD). Our investigation identified 19 bacterial genera and associated metabolic pathways linked to IPF (Supplementary Table 1), alongside 21 bacterial genera and metabolic pathways associated with COPD (Supplementary Table 2). By intersecting these findings, we discerned the presence of an aspartate superpathway within the metabolic repertoire of gut microbiota relevant to both IPF and COPD. Subsequently, this discovery served as a focal point for a follow-up study (Supplementary Figure 1).

### MR analysis of the gut microbiota metabolic pathways

**Figure 2:**
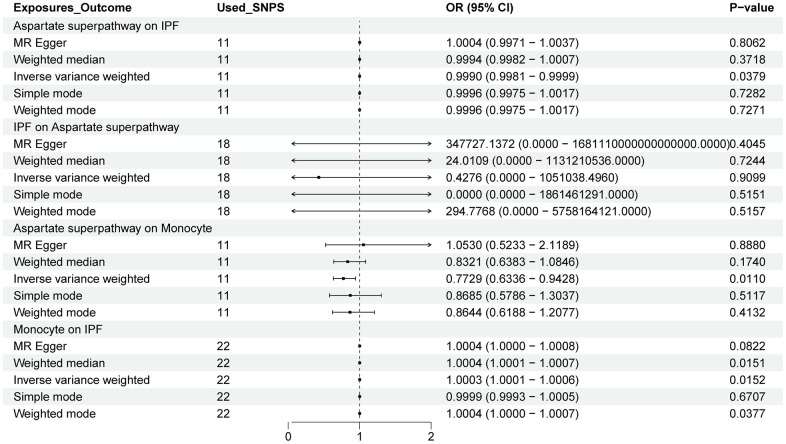
**The forest plot shows the causal relationship between the aspartate superpathway, monocytes, and IPF.** The relationship between the exposure variable and the outcome is calculated through several functions, including MR Egger, weighted median, inverse variance weighted (IVW), simple mode, and weighted mode. Among them, IVW is the primary method. OR: odds ratio; CI: confidence interval.

**Figure 3:**
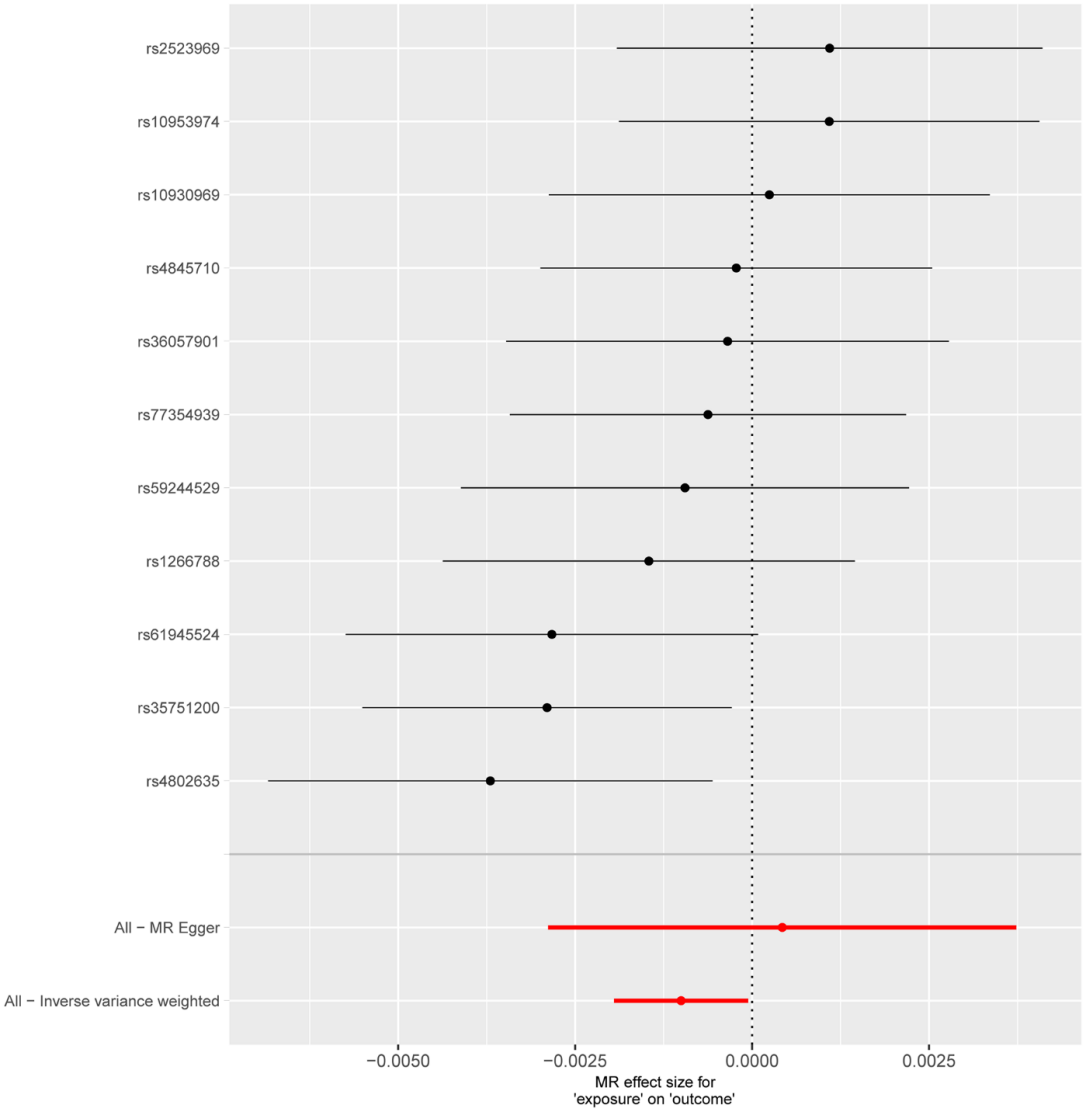
**Forest plot shows the causal effect of each single SNP on total IPF risk.** The horizontal axis is employed to quantify the magnitude of the impact exerted by the exposure factor on the outcome, whereas the vertical axis denotes single-nucleotide polymorphisms (SNPs). A negative value implies that the exposure may decrease the risk of the outcome’s occurrence, while a positive value suggests that the exposure may elevate the risk of the outcome’s occurrence. The inverse-variance weighted (IVW) method is utilized to assess validity, and the Mendelian randomization Egger (MR Egger) method is applied to detect and correct for potential horizontal pleiotropy.

We identified 11 Single Nucleotide Polymorphisms (SNPs) as Instrumental Variables (IVs) based on our screening criteria. Utilizing the inverse variance weighted (IVW) algorithm, our analysis revealed that the aspartate super pathway posed a protective factor for Idiopathic Pulmonary Fibrosis (IPF) (*p-value*=0.0379, Odds Ratio=0.999) (Figure 2). Furthermore, both the Mendelian randomization-Egger (MR-Egger) and IVW assays indicated no significant heterogeneity in our results (Figure 3).

**Figure 4:**
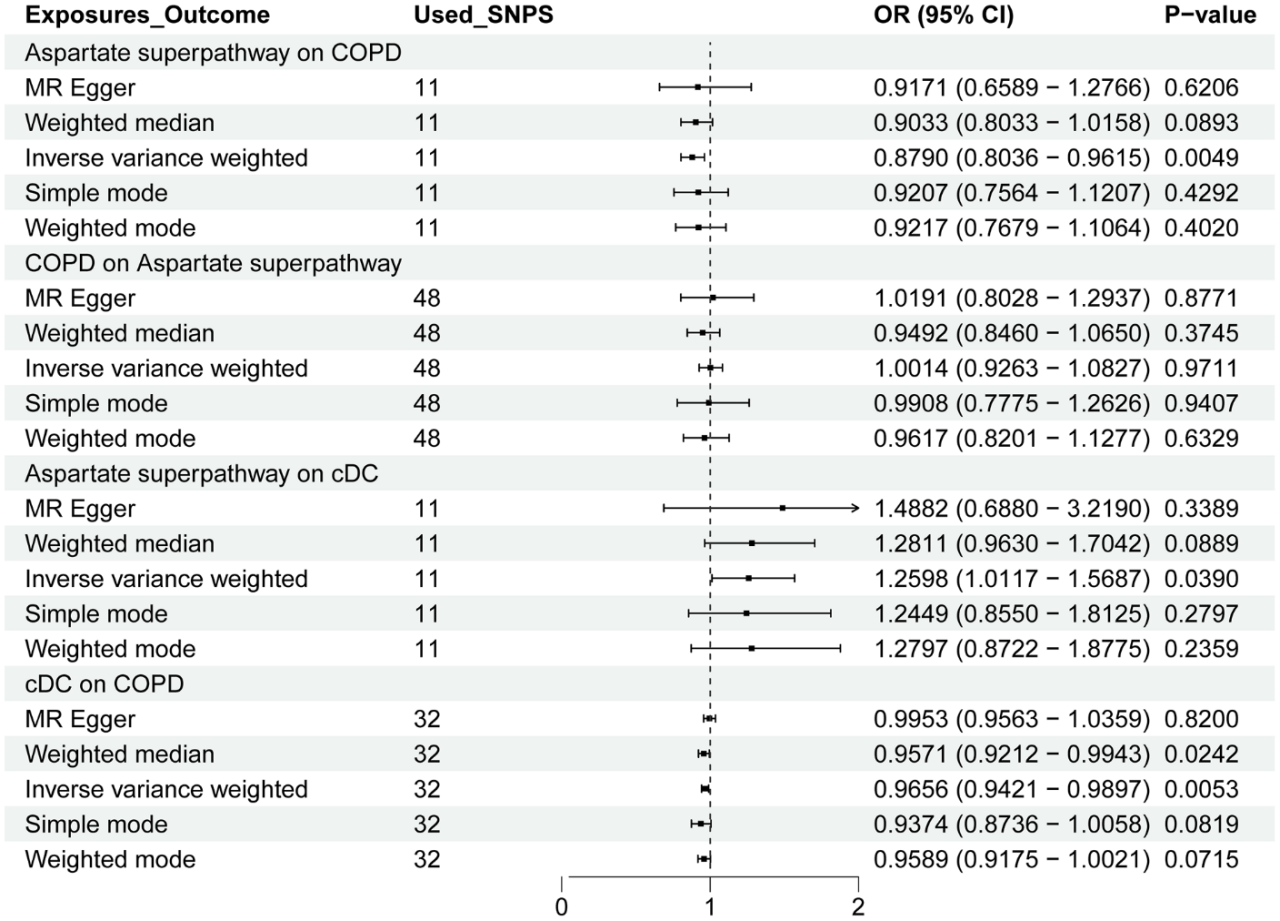
**The forest plot shows the causal relationship between the aspartate superpathway, cDC, and COPD.** OR: odds ratio; CI: confidence interval. The relationship between the exposure variable and the outcome is calculated through several functions, including MR Egger, weighted median, inverse variance weighted (IVW), simple mode, and weighted mode. Among them, IVW is the primary method.

**Figure 5:**
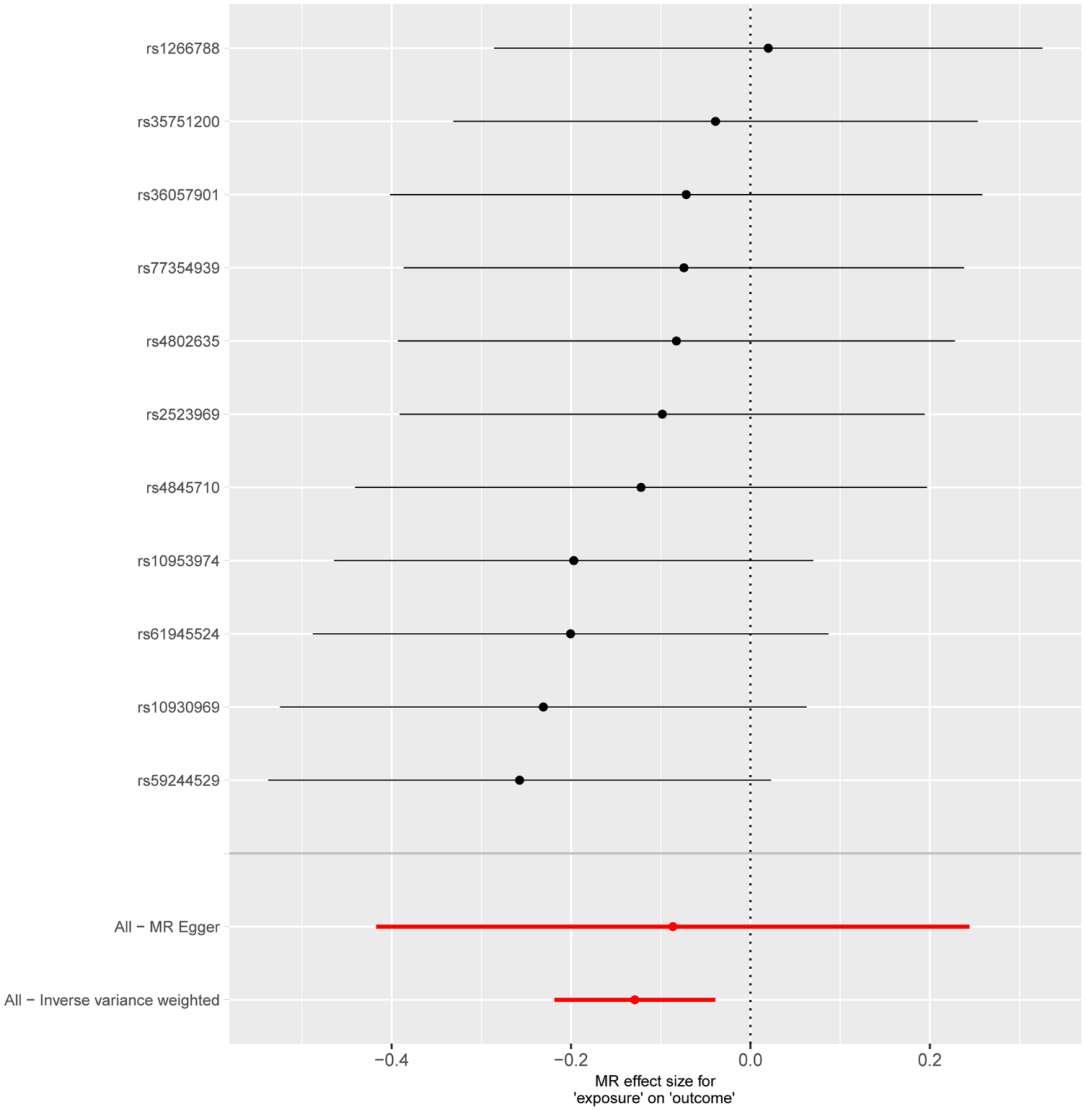
**Forest plot showing the causal effect of each single SNP on total COPD risk.** The horizontal axis is employed to quantify the magnitude of the impact exerted by the exposure factor on the outcome, whereas the vertical axis denotes single-nucleotide polymorphisms (SNPs). A negative value implies that the exposure may decrease the risk of the outcome’s occurrence, while a positive value suggests that the exposure may elevate the risk of the outcome’s occurrence. The inverse-variance weighted (IVW) method is utilized to assess validity, and the Mendelian randomization Egger (MR Egger) method is applied to detect and correct for potential horizontal pleiotropy.

Following a predefined screening protocol and after excluding confounding factors and irrelevant SNPs, we identified 11 SNPs for further analysis. The Mendelian Randomization (MR) analysis indicated that the aspartate superpathway constitutes a protective factor for Chronic Obstructive Pulmonary Disease (COPD). Notably, there was a statistically significant difference between the results obtained from different IVW algorithms (*p-value* < 0.05). However, the remaining four algorithms exhibited consistent directional effects. Specifically, the IVW analysis yielded an odds ratio (OR) of 0.879, with a 95% confidence interval (CI) of 0.8036-0.9615 (Figure 4). Detection of horizontal pleiotropy and heterogeneity was further confirmed through MR-Egger and IVW analyses (Figure 5).

### Screening of immune cells

We conducted reverse Mendelian Randomization (MR) analysis using Idiopathic Pulmonary Fibrosis (IPF) and Chronic Obstructive Pulmonary Disease (COPD) as exposure factors and the aspartate super pathway as the outcome. The findings revealed no discernible causal relationship between IPF and the aspartate super pathway, similar results were obtained for COPD. These outcomes suggest the potential involvement of intermediary factors in the intricate interaction between gut microbiota and the development of IPF and COPD.

Given the close association of diseases with immune responses, we explored correlations between IPF, COPD, and immune cell populations. Our estimates demonstrated a robust correlation between IPF and monocytes (specifically, HLA DR expression on CD14+ monocytes), consistent with findings reported in previous studies. Similarly, for COPD, the analysis indicated a correlation with conventional dendritic cells (cDCs) expressing CD80. These findings shed light on potential immune-mediated mechanisms underlying the pathogenesis of IPF and COPD.

### MR analysis of immune cells

The aspartate superpathway served as an exposure factor to investigate the causal relationship with two distinct outcome variables: monocytes and cDC. Leveraging the 11 SNPs identified through screening as instrumental variables (IVs) for Mendelian Randomization (MR) analysis, our results indicated significant associations. Specifically, employing the IVW algorithm, we observed a statistically significant association (p-value < 0.05) between the aspartate superpathway and monocytes, suggesting a protective factor effect with an odds ratio (OR) of 0.772 (Figure 2). Conversely, for cDC, the IVW algorithm yielded a significant association (p-value < 0.05) indicating a risk effect with an OR of 1.260 (Figure 4).

Furthermore, employing MR analysis to explore the causal link between immune cells and disease, we focused on the relationships with Idiopathic Pulmonary Fibrosis (IPF) and Chronic Obstructive Pulmonary Disease (COPD). For IPF, screening identified 22 monocyte-associated SNPs as IVs. Notably, the IVW algorithm revealed a significant risk effect of monocytes against IPF (p-value < 0.05, OR = 1.0003) (Figure 2). Conversely, in the context of COPD, employing 32 cDC-related IVs, the IVW analysis showed a significant association (p-value < 0.05, OR = 0.966), indicating that cDC represents a protective factor for COPD (Figure 4).

### Analysis of mediating effects

Based on the two-sample bidirectional mediation analysis, we have derived the following hypotheses.

1. The aspartate super pathway participates in the protective effects of IPF by inhibiting monocytes, in which monocytes induce IPF development.
2. The aspartate super pathway participates in the protective effects of COPD by enhancing the involvement of cDC, and cDC reduces the incidence of COPD.

Our mediation effect analysis revealed specific insights. For IPF, the direct effect of the aspartate super pathway was calculated as -0.0009240468. Additionally, the analysis indicated that 7.88% of the effect was mediated through monocytes (direct effect=-7.908946e-05). Conversely, in the context of COPD, the direct effect of the aspartate super pathway was -0.1208708. Notably, 6.27% of this effect was mediated via cDC (with an indirect effect of 0.81%).

## Discussion and Conclusions

Once perceived merely as a digestive organ, the gastrointestinal tract has undergone a renaissance in scientific exploration, with advancements in technology unveiling the profound impact of gut microbiota on human health and disease [18]. The ubiquitous presence of microbes across many organs in the human body suggests potential inter-organ flora interactions. The proposition of the “lung-gut axis” has sparked novel inquiries into the potential influence of gut microbiota on chronic respiratory ailments prevalent in the elderly [19]. In our study, we meticulously screened genera and metabolic pathways associated with Idiopathic Pulmonary Fibrosis (IPF) and Chronic Obstructive Pulmonary Disease (COPD), identifying the aspartate superpathway through intersectional analysis. This pathway encompasses the synthesis of various compounds (Supplementary Table 3, https://biocyc.org/), including amino acids such as L-aspartate, lysine, threonine, and methionine, along with coenzymes such as S-adenosylmethionine (SAM) and nicotinamide adenine dinucleotide (NAD) [20]. The clinical significance of the aspartate superpathway is mainly reflected in cancer, neurological diseases, and cardiovascular diseases. In the treatment of acute lymphoblastic leukemia, asparaginase is used as a chemotherapy agent to degrade extracellular asparagine, thereby inhibiting the growth of leukemia cells. Aspartate can also be converted to glutamate through transamination. Glutamate is essential for the synthesis of glutathione, a key antioxidant that scavenges free radicals in nerve cells and reduces oxidative stress. In neurodegenerative diseases such as Parkinson’s disease, metabolic abnormalities in the aspartate superpathway may impair the synthesis of glutathione, thereby weakening the antioxidant capacity of nerve cells and accelerating disease progression. Similarly, in cardiovascular diseases such as hypertension and atherosclerosis, disruptions in the aspartate superpathway can affect the arginine-NO pathway, leading to vascular endothelial dysfunction and promoting disease development.

IPF and COPD are both chronic, progressive, and heterogeneous respiratory diseases that can culminate in deteriorating lung function and respiratory failure [21]. Emerging research underscores abnormal organismal repair mechanisms underlying IPF and COPD pathogenesis. The etiology of these diseases remains elusive, although genetic predisposition, telomere shortening, and mitochondrial damage are implicated [22]. Animal models suggest that lifestyle and dietary factors can modulate gut microbiota composition, thereby influencing pulmonary fibrosis progression. Perturbations in glucose-lipid metabolism observed in IPF patients highlight the potential association of gut microbiota metabolic pathways with disease progression [23]. Antibiotic and probiotic interventions in COPD treatment exhibit promising outcomes by enhancing lung immunity and restoring gut microbiota balance, albeit requiring further mechanistic elucidation. Increased D-Asp residues in COPD patients exacerbate mitochondrial damage, accentuating the potential role of aspartic acid in common elderly chronic respiratory diseases [24].

Recent insights underscore the intricate involvement of gut microbiota in immune processes through modulation of metabolic pathways like short-chain fatty acids, bile acids, and tryptophan, with ramifications on distal organs [25]. Monocytes, as innate immune cells expressing multiple microbial receptors, play pivotal roles in colitis [26] and cancer [27] through dectin-1 and STING pathway activation by gut microbiota ligands [28]. Our study corroborates the significant role of monocytes in IPF development, mediated by gut microbiota metabolic pathways [29]. Dendritic cells, which are proficient antigen-presenting cells crucial in T cell-mediated immune responses, exhibit elevated quinolinate expression after LPS stimulation. It is worth noting that this metabolite is associated with the central metabolite oxalacetic acid in the aspartate superpathway, providing a potential mechanism for our finding [30].

In summary, our study identified that the alteration of gut microbiota is related to the onset of IPF and COPD. Specifically, metabolites that result from the aspartate superpathway of the gut microbiota can influence immune cells including monocytes and cDCs, which will subsequently function in IPF and COPD pathogenesis.

Despite our study’s robustness, certain limitations warrant acknowledgment. The sample population is mainly composed of European cohorts. It may not comprehensively and accurately reflect the inherent characteristics, genetic profiles, and responses of other diverse ethnic and geographical populations. Experimental validation is warranted to complement our retrospective analysis, urging a longitudinal perspective. Nevertheless, our study underscores the causal association of the aspartate superpathway with COPD and IPF using Mendelian randomization, highlighting immune cells as potential mediators influencing disease trajectory and offering innovative therapeutic avenues for COPD and IPF management.

## Supplementary Material

Supplementary Figure 1

Supplementary Tables
