# Supplementary material for: The aspartate superpathway in gut microbiota-related metabolic pathways mediates immune cell protection against COPD and IPF: a Mendelian randomization analysis
Source: Aging (Albany NY). 2025 May 15;17(5):1298–312. doi: 10.18632/aging.206250 (PMC12151505; doi:10.18632/aging.206250)
Supplement: Supplementary Figure 1 [file aging-17-206250-s001.pdf]

SUPPLEMENTARY FIGURE

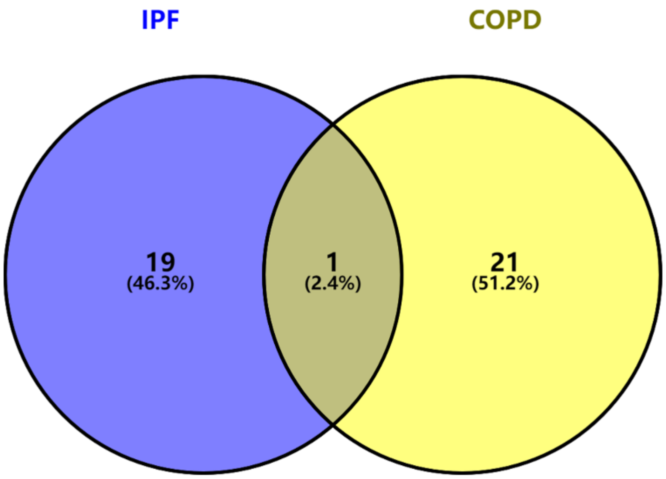

Supplementary Figure 1. Venn diagram showing the numbers of IPF- and COPD-associated gut microbiota pathways. The aspartate superpathway (shown by the intersection) is a microbiota metabolic pathway associated with both IPF and COPD. 19 IPF-associated and 21 COPD-associated gut microbiota composition were taken to be intersected.
