# Supplementary material for: The aspartate superpathway in gut microbiota-related metabolic pathways mediates immune cell protection against COPD and IPF: a Mendelian randomization analysis
Source: Aging (Albany NY). 2025 May 15;17(5):1298–312. doi: 10.18632/aging.206250 (PMC12151505; doi:10.18632/aging.206250)
Supplement: Supplementary Tables [file aging-17-206250-s002.pdf]

SUPPLEMENTARY TABLES

Supplementary Table 1. IPF-related gut microbiota and metabolic pathways.

id	name	*p*-value
GCST90027459	DAPLYSINESYN.PWY..L.lysine.biosynthesis.I	0.0174982873746744
GCST90027476	GLYCOGENSYNTH.PWY..glycogen.biosynthesis.I..from.ADP.D.Glucose.	0.0258085976949285
GCST90027520	PWY0.1338..polymyxin.resistance	0.000561305378200455
GCST90027526	PWY0.781..aspartate.superpathway	0.0378528122088573
GCST90027537	PWY.5022..4.aminobutanoate.degradation.V	0.0268849216334508
GCST90027541	PWY.5101..L.isoleucine.biosynthesis.II	0.0381510042823094
GCST90027562	PWY.5918..superpathay.of.heme.biosynthesis.from.glutamate	0.0321507133378937
GCST90027590	PWY.6629..superpathway.of.L.tryptophan.biosynthesis	0.00992156423579139
GCST90027593	PWY.6690..cinnamate.and.3.hydroxycinnamate.degradation.to.2.oxopent.4.enoate	0.00668720180700082
GCST90027609	PWY.7197..pyrimidine.deoxyribonucleotide.phosphorylation	0.0482522745688315
GCST90027710	k_Bacteria.p_Firmicutes.c_Clostridia.o_Clostridiales.f_Lachnospiraceae.g_Coprococcus	0.0448015037701725
GCST90027724	k_Bacteria.p_Firmicutes.c_Negativicutes.o_Selenomonadales.f_Veillonellaceae.g_Veillonella	0.0211839781922249
GCST90027751	k_Bacteria.p_Proteobacteria	0.00766392094032009
GCST90027767	k_Bacteria.p_Bacteroidetes.c_Bacteroidia.o_Bacteroidales.f_Porphyromonadaceae.g_Parabacteroides.s_Parabacteroides_goldsteinii	0.0349290697876317
GCST90027773	k_Bacteria.p_Bacteroidetes.c_Bacteroidia.o_Bacteroidales.f_Prevotellaceae.g_Paraprevotella.s_Paraprevotella_xylaniphila	0.0248353543722653
GCST90027782	k_Bacteria.p_Firmicutes.c_Bacilli.o_Lactobacillales.f_Lactobacillaceae.g_Lactobacillus.s_Lactobacillus_delbrueckii	0.0128776814690116
GCST90027803	k_Bacteria.p_Firmicutes.c_Erysipelotrichia.o_Erysipelotrichales.f_Erysipelotrichaceae.g_Erysipelotrichaceae_noname.s_Eubacterium_biforme	0.0123723137486221
GCST90027805	k_Bacteria.p_Firmicutes.c_Erysipelotrichia.o_Erysipelotrichales.f_Erysipelotrichaceae.g_Holdemania.s_Holdemania_unclassified	0.0334695199585892
GCST90027822	k_Bacteria.p_Bacteroidetes.c_Bacteroidia.o_Bacteroidales.f_Bacteroidaceae.g_Bacteroides.s_Bacteroides_clarus	0.0372791341579532
GCST90027850	k_Bacteria.p_Firmicutes.c_Clostridia.o_Clostridiales.f_Lachnospiraceae.g_Lachnospiraceae_noname.s_Lachnospiraceae_bacterium_3_1_46FAA	0.0103334164682713

Statistical significance was considered at $p < 0.05$.

Supplementary Table 2. COPD-related gut microbiota and metabolic pathways.

id	name	*p*-value
GCST90027454	COA.PWY..coenzyme.A.biosynthesis.I	0.0456156517859008
GCST90027458	CRNFORCAT.PWY..creatinine.degradation.I	0.0450799236171167
GCST90027460	DENOVOPURINE2.PWY..superpathway.of.purine.nucleotides.de.novo.biosynthesis.II	0.0260102805544053
GCST90027501	P42.PWY..incomplete.reductive.TCA.cycle	0.00164234790594733
GCST90027526	PWY0.781..aspartate.superpathway	0.00485565879440769
GCST90027538	PWY.5088..L.glutamate.degradation.VIII..to.propanoate.	0.0330992473218536
GCST90027543	PWY.5121..superpathway.of.geranylgeranyl.diphosphate.biosynthesis.II..via.MEP.	0.00763686025662704
GCST90027585	PWY.6590..superpathway.of.Clostridium.acetobutylicum.acidogenic.fermentation	0.0179446292169535
GCST90027632	PWY_GLYOXYLATE.BYPASS..glyoxylate.cycle	0.0287543285399453
GCST90027689	k_Bacteria.p_Actinobacteria.c_Actinobacteria.o_Coriobacteriales.f_Coriobacteriaceae.g_Adlercreutzia	0.0346146070242656
GCST90027703	k_Bacteria.p_Firmicutes.c_Bacilli.o_Lactobacillales.f_Streptococcaceae.g_Streptococcus	0.0348109428077718
GCST90027705	k_Bacteria.p_Firmicutes.c_Clostridia.o_Clostridiales.f_Clostridiales_noname.g_Flavonifractor	0.0110893986035683
GCST90027758	k_Bacteria.p_Actinobacteria.c_Actinobacteria.o_Coriobacteriales.f_Coriobacteriaceae.g_Adlercreutzia.s_Adlercreutzia_equolifaciens	0.0348423554504129
GCST90027774	k_Bacteria.p_Bacteroidetes.c_Bacteroidia.o_Bacteroidales.f_Prevotellaceae.g_Prevotella.s_Prevotella_copri	0.0275518765301815
GCST90027790	k_Bacteria.p_Firmicutes.c_Clostridia.o_Clostridiales.f_Eubacteriaceae.g_Eubacterium.s_Eubacterium_eligens	0.0224288748777411
GCST90027808	k_Bacteria.p_Firmicutes.c_Negativicutes.o_Selenomonadales.f_Veillonellaceae.g_Veillonella.s_Veillonella_unclassified	0.0469163857617631
GCST90027817	k_Bacteria.p_Proteobacteria.c_Gammaproteobacteria.o_Enterobacteriales.f_Enterobacteriaceae.g_Escherichia.s_Escherichia_unclassified	0.0374646593397856
GCST90027824	k_Bacteria.p_Bacteroidetes.c_Bacteroidia.o_Bacteroidales.f_Bacteroidaceae.g_Bacteroides.s_Bacteroides_dorei	0.0116210412375593
GCST90027842	k_Bacteria.p_Firmicutes.c_Clostridia.o_Clostridiales.f_Lachnospiraceae.g_Butyrivibrio.s_Butyrivibrio_crossotus	0.0411945185417917
GCST90027845	k_Bacteria.p_Firmicutes.c_Clostridia.o_Clostridiales.f_Lachnospiraceae.g_Coprococcus.s_Coprococcus_sp_ART55_1	0.0185148218336091
GCST90027848	k_Bacteria.p_Firmicutes.c_Clostridia.o_Clostridiales.f_Lachnospiraceae.g_Dorea.s_Dorea_unclassified	0.0179394856149212
GCST90027852	k_Bacteria.p_Firmicutes.c_Clostridia.o_Clostridiales.f_Lachnospiraceae.g_Lachnospiraceae_noname.s_Lachnospiraceae_bacterium_7_1_58FAA	0.021748490257721

Statistical significance was considered at $p < 0.05$.

Supplementary Table 3. Major components of the aspartate superpathway.

MetaCyc-Main compound
oxaloacetate
L-aspartate
2-iminosuccinate
quinolinate
β-nicotinate D-ribonucleotide
nicotinate adenine dinucleotide
NAD
L-aspartyl-4-phosphate
L-aspartate 4-semialdehyde
(2S,4S)-4-hydroxy-2,3,4,5tetrahydrodipicolinate
(S)-2,3,4,5-tetrahydrodipicolinate
N-succinyl-2-amino-6-ketopimelate
N-succinyl-L,L-2,6-diaminopimelate
L,L-diaminopimelate
meso-diaminopimelate
L-lysine
L-homoserine
L-cysteine
O-succinyl-L-homoserine
O-phospho-L-homoserine
L-threonine
L-cystathionine
L-homocysteine
L-methionine
S-adenosyl-L-methionine

Retrieved from https://biocyc.org/.
